# Outbreak of suspected Clostridium butyricum botulism in India.

**DOI:** 10.3201/eid0403.980347

**Published:** 1998

**Authors:** R Chaudhry, B Dhawan, D Kumar, R Bhatia, J C Gandhi, R K Patel, B C Purohit


### Outbreak of Suspected *Clostridium butyricum* Botulism in India

**To the Editor:** Foodborne botulism, particularly associated with *Clostridium butyricum,* is rare; no cases had been reported in India before this outbreak. A reported case of foodborne botulism represents a public health emergency because of the potential severity of the disease and the possibility of mass exposure to the contaminated product.

In September 1996, the anaerobic section of the All India Institute of Medical Sciences received serum and food samples from the National Institute of Communicable Diseases, Delhi, India, for investigating a possible outbreak of foodborne botulism.

In the early hours of September 18, 1996, 34 of 310 students of a residential school in rural Gujrat complained of abdominal pain, nausea, chest pain, and difficulty in breathing. One of the students, aged 14, died before he could be treated; two others, aged 13, died on their way to the hospital. The remaining 31 students were admitted to a rural hospital; eight were discharged 1 day later after being given symptomatic treatment, while the other 23 were transported by ambulance to an urban emergency department in Ahemdabad, Gujrat. Findings on examination included ptosis, pupillary mydriasis, extraocular palsies, and impairment of conciousness. All students were given symptomatic treatment in the form of stomach lavage and intravenous administration of antibiotics and steroids. Over the subsequent 24 hours, 21 improved clinically and were discharged; however, two (aged 14 and 17 years) had respiratory distress and required mechanical ventilation. Differential diagnosis included botulinum food poisoning, and both patients were administered trivalent (A,B,E) botulinum antitoxin. They responded well to the treatment and were discharged from the hospital 1 month later.

Patients reported that 24 hours before onset of symptoms, they had eaten ladoo (a local sweet), curd, buttermilk, sevu (crisp made of gram flour), and pickle. Food samples were assayed for botulinum toxin and were cultured anaerobically (1). Anaerobic culture of leftover sevu yielded an organism in pure culture whose cultural and biochemical properties were consistent with those of *C. butyricum;* i.e., it was lipase-negative, fermentative, and did not liquefy gelatin (2). Enrichment cultures of the sevu specimens in enriched chopped meat–glucose–starch medium contained toxin after 5 days of anaerobic incubation at 30°C. This was shown by mouse toxicity test in which the enrichment broth of the specimen was injected intraperitoneally into mice; botulinum toxin was detected by observing its lethal effect on mice. This effect was neutralized by specific polyvalent botulinum antitoxin types A, B, E (Biomed, Warsaw, Poland). Cultures of other food items tested negative for toxigenic organisms. Serum specimens (obtained more than 1 week after the onset of illness) from eight patients with mildly symptomatic illness were negative for toxin.

To test the presence of toxin gene in the isolated strain of *C. butyricum*, polymerase chain reaction (PCR) was performed. Degenerate primers BoNT 1 and BoNT 2 were used, which amplify a specific 1.1-kb fragment of neurotoxin gene *C. botulinum* types (A, B, E, F, and G) as well as toxigenic strains of *C. baratti* and *C. butyricum* (3). Five *Escherichia coli* strains containing clones encoding fragments of the *C. botulinum* neurotoxin genes were used as positive controls in the PCR assay (kindly provided by Alison East, Institute of Food Research, United Kingdom). PCR profile used was as follows: 94°C for 2 min, followed by 25 cycles of 92°C for 1 min, 42°C for 1 min, and 62°C for 5 min, then held at 4°C (Alison East, pers. comm.). An amplified product of 1.1 kb was detected from the culture isolate of sevu.

The outbreak described in this report draws attention to the emergence of new foodborne pathogens and to their association with unusual foods. Human botulism is commonly caused by *C. botulinum* neurotoxin type A, B, and E (4). In the present study, we showed that a neurotoxigenic *C. butyricum* was present in the food implicated in a clinically suspected outbreak of botulism in Gujrat, India.

Laboratory studies could not confirm the diagnosis of botulism because clinical materials (such as contents of the gastrointestinal tract, feces) were not submitted for examination for the presence of the botulinum toxin or organisms. It is not surprising that toxin could not be detected in the eight serum samples received by our laboratory. Because of the delay in clinical diagnosis, early serum samples could not be obtained. Toxin is detected in only 13% of serum samples collected more than 2 days after ingestion of botulinum toxin (5). However, the clinical presentation of the patients, response to trivalent botulinum antitoxin, and isolation of toxigenic *C. butyricum* from one of the consumed food articles strongly suggest that the outbreak was caused by food contaminated with toxigenic *C. butyricum*.

Neurotoxigenic *C. butyricum* was first reported in 1986 in two cases of infant botulism in Rome (6). Recently, neurotoxigenic *C. butyricum* was isolated from the food implicated in an outbreak of clinically diagnosed type E botulism in China (7). In this outbreak, it appears that sevu, because of improper storage, was contaminated with the spores of *C. butyricum*, which subsequently germinated and produced toxin. To the best of our knowledge, this is the first report of neurotoxigenic *C. butyricum* causing foodborne botulism in India.

The changing epidemiology of foodborne disease as highlighted in this report calls for improved surveillance, including the development of new technology for identifying outbreaks.

We thank Alison East, Institute of Food Research, Reading Laboratory, United Kingdom, for supplying *E. coli* clones with BONT gene for PCR; Pradeep Seth, professor and head, Department of Microbiology, All India Institute of Medical Sciences for facilities provided; Biomed Warsaw, Poland, for polyvalent botulinum antitoxin; and the medical and paramedical staff of Civil Hospital, Ahemdabad.

**Rama Chaudhry,* Benu Dhawan,* Dinesh Kumar,* Rajesh Bhatia,† J.C Gandhi,‡ R.K. Patel,§ and B.C. Purohit§**

All India Institute of Medical Sciences, New Delhi, India; †National Institute of Communicable Diseases, Delhi, India; ‡Health Medical Services and Medical Education (H.S.), Gandhi Nagar, India; and §Civil Hospital, Ahemdabad, Gujrat, India

### References

1. Hatheway CL. Botulism. In: Balows A, Hausler WJ, Lennette EH, editors. Laboratory diagnosis of infectious diseases: principles and practice. New York: Springer-Verlag: 1988. p. 111-33.
2. McCroskey LM, Hatheway CL, Fenicia L, Pasolini B, Aureli P. Characterization of an organism that produces type E botulinal toxin but which resembles *Clostridium butyricum* from the feces of an infant with type E botulism. J Clin Microbiol 1986;23:201-2.
3. Campbell KD, Collins MD, East AK. Gene probes for identification of the Botulinal Neurotoxin gene and specific identification of neurotoxin types B.E. and F.J. Clin Microbiol 1993;31:2255-62.
4. Hatheway CL. *Clostridium botulinum* and other clostridia that produce botulinum neurotoxin. In: Hauschild AHW, Dodds KL, editors. *Clostridium botulinum*—ecology and control in foods. New York: Marcel Dekker, Inc.; 1992. p. 3-20.
5. Woodruff BA, Griffin PM, McCroskey LM, Smart JF, Wainwright RB, Bryant RG, et al. Clinical and laboratory comparison of botulism from toxin types A, B, and E in the United States, 1975-1988. J Infect Dis 1992;166:1281-6.
6. Aureli PK, Fenicia L, Pasolini B, Gianfranceschi M, McCroskey LM, Hatheway CL. VII. Two cases of type E infant botulism caused by neurotoxigenic *Clostridium butyricum* in Italy. J Infect Dis 1986;154:207-11.
7. Meng X, Karasawa T, Zou K, Kuang X, Wang X, Lu C. et al. Characterization of a neurotoxigenic *Clostridium butyricum* strain isolated from the food implicated in an outbreak of food-borne type E botulism. J Clin Microbiol 1997;35:2160-2.

### Molecular Analysis of *Salmonella paratyphi* A From an Outbreak in New Delhi, India

**To the Editor:** In the context of emerging infectious diseases, enteric fever caused by *Salmonella paratyphi* A deserves increased attention and vigilance, although its severity is often milder than that of *S. typhi* disease. Outbreaks associated with this organism are exceedingly rare but have recently been reported in India (1) and Thailand. In India, the first reported outbreak of disease associated with *S. paratyphi* A (1) provided an opportunity to study the molecular epidemiology of infection caused by this organism.

A total of 18 human blood isolates of *S. paratyphi* A, 13 from the outbreak in New Delhi, India (from September to October 1996) (1) and 5 sporadic isolates from cases unrelated to the outbreak, were used in this study. A total of 36 culture-positive cases were detected during the 6-week outbreak. All strains were phage type 1 and were sensitive to all antibiotics tested. Isolates were analyzed by ribotyping and pulsed-field gel electrophoresis (PFGE) (2,3). PFGE/ribotype profiles were assigned arbitrary designations and analyzed by defining a similarity (Dice) coefficient, F (3), where F = 1.0 indicates complete pattern identity and F = 0, complete dissimilarity.

The five sporadic isolates of *S. paratyphi* A gave PFGE patterns following *Xba*I (5'-TCTAGA-3') digestion that were unique and distinctly different, with differences of 8 to 12 bands (F = 0.63-0.70). In contrast, the 13 outbreak isolates shared only four closely related PFGE patterns differing only in 1 to 6 bands (F = 0.8-1.0). Among the outbreak strains, two distinct clones were

